# StAR: a simple tool for the statistical comparison of ROC curves

**DOI:** 10.1186/1471-2105-9-265

**Published:** 2008-06-05

**Authors:** Ismael A Vergara, Tomás Norambuena, Evandro Ferrada, Alex W Slater, Francisco Melo

**Affiliations:** 1 Departamento de Genética Molecular y Microbiología, Facultad de Ciencias Biológicas, Pontificia Universidad Católica de Chile, Alameda 340, Santiago, Chile

## Abstract

**Background:**

As in many different areas of science and technology, most important problems in bioinformatics rely on the proper development and assessment of binary classifiers. A generalized assessment of the performance of binary classifiers is typically carried out through the analysis of their receiver operating characteristic (ROC) curves. The area under the ROC curve (AUC) constitutes a popular indicator of the performance of a binary classifier. However, the assessment of the statistical significance of the difference between any two classifiers based on this measure is not a straightforward task, since not many freely available tools exist. Most existing software is either not free, difficult to use or not easy to automate when a comparative assessment of the performance of many binary classifiers is intended. This constitutes the typical scenario for the optimization of parameters when developing new classifiers and also for their performance validation through the comparison to previous art.

**Results:**

In this work we describe and release new software to assess the statistical significance of the observed difference between the AUCs of any two classifiers for a common task estimated from paired data or unpaired balanced data. The software is able to perform a pairwise comparison of many classifiers in a single run, without requiring any expert or advanced knowledge to use it. The software relies on a non-parametric test for the difference of the AUCs that accounts for the correlation of the ROC curves. The results are displayed graphically and can be easily customized by the user. A human-readable report is generated and the complete data resulting from the analysis are also available for download, which can be used for further analysis with other software. The software is released as a web server that can be used in any client platform and also as a standalone application for the Linux operating system.

**Conclusion:**

A new software for the statistical comparison of ROC curves is released here as a web server and also as standalone software for the LINUX operating system.

## Background

The prediction of discrete states or categories for any event or for any object requires a classification process. In order to be useful, many real-world applications rely on an optimized classification process. These include some important problems such as diagnosis of diseases, definition of medical treatments, economical and security risk assessment, weather forecast, air traffic regulation and quality control of industrial processes [1].

Classification tasks in bioinformatics are also common and can be found in many different and relevant applications, such as the prediction of genome and protein structure [2,3], the prediction of the cellular location [4], the prediction of molecular function [5] and the prediction of molecular interactions [6]. In general, a classification process is always involved in the prediction of a pattern that can be related to some response in living systems.

A popular approach for assessing binary classifiers is analysis of their ROC curves on a set of representative data [7,8]. A ROC curve corresponds to a bidimensional plot of the sensitivity versus 1-specificity for a given classifier with continuous or ordinal output score. Two main factors have to be considered by the user when estimating the ROC curves: **1) The design of the study**. Three types of dataset can be generated when pursuing a classification task: (i) paired data, where all classifiers are applied to each individual, (ii) unpaired data, where only one of the classifiers is applied to each individual, and (iii) partially-paired data [9], where the dataset is composed of both paired and unpaired data. In the case of paired and partially-paired datasets, correlation between ROC curves has to be taken into consideration. Our software is primarily designed for paired data. However, it can also analyze balanced unpaired data where the number of units is the same for each classifier. It cannot be used with partially-paired data. **2) The outcome distribution**. Depending on this factor, three types of approaches can be more or less appropriate for fitting ROC curves and estimating the corresponding AUCs: (i) A parametric approach [10,11], where we assume a parametric distribution for the outcomes of the positive and negative individuals. (ii) A semiparametric approach, where we assume that discrete ordinal outcomes correspond to classification of an unobserved latent decision variable into ordinal categories defined by unknown cut-points or threshold values, or that continuous outcomes can be expressed as an unknown monotonic transformation of the latent distribution [12], with positive and negative individuals having different latent decision variables. In this case, parametric distributions (e.g. normal, logistic, log-normal) are assumed for the latent decision variables. (iii) A non-parametric approach [13,14], where no distributional assumptions are made about the outcomes for the positive and negative individuals.

For each type of approach, different methods for estimating the AUC after the ROC curve is generated have been described [11,15,16]. The advantages and disadvantages of using one or another approach under different scenarios have been previously assessed [17,18]. Among the advantages of using a parametric or semiparametric method are that these methods generate a smooth ROC curve, and the assumption of a distribution provides a natural means by which statistical inference such as hypothesis testing and confidence intervals can be achieved. When data deviate from the assumed distribution (e.g. normal or log-normal) or simply the outcome distribution for the positive or negative individuals is uncertain, non-parametric methods for estimating the ROC curve become a useful and robust alternative. Even though the ROC curve generated by non-parametric methods is jagged, that problem has been tackled in a non-parametric manner by means of kernel density estimation of the empirical distributions in a previous study [13].

Although many examples in the existing literature about the development of new classifiers describe the use of ROC curves and their corresponding AUCs to assess their performances, the statistical significance of their differences is often not reported. This is mostly due to the lack of freely available software that is easy to use or to automate for the pairwise comparison of many binary classifiers. Albeit there are several software for performing statistical ROC analysis [19], to the best of our knowledge, the only free and readily available software for statistical ROC analysis that assesses the significance of the difference of the AUC for a pair of classifiers is ROCKIT [20,21]. This software uses maximum likelihood to fit a binormal ROC curve to the data and the statistical significance of the differences of a variety of indexes are assessed on the basis of a bivariate binormal model. In terms of usability, it has some drawbacks: 1) the input data format is rather cumbersome; 2) the output file contains many relevant data embedded in a human-readable text and thus needs to be parsed for further analysis; 3) the number of classifiers that can be simultaneously assessed is quite limited; 4) additional software is needed for plotting the ROC curves; 5) in case of errors, the program does not provide any feedback to the user about the causes of the abnormal interruption; and 6) it cannot be easily automated when a fast comparison of several classifiers is required.

In this work we describe new software that is freely available as a web server tool and also as a standalone application for the Linux operating system that allows the simultaneous pairwise comparison and statistical assessment of many binary classifiers. The approach chosen is the nonparametric method for comparing AUCs based on the Mann-Whitney U-statistic for comparing distributions of values from two samples [14]. It has been shown that the AUC calculated by the trapezoidal rule is equal to the Mann-Whitney U-statistic applied to the outcomes for the negative and positive individuals. Thus, two or more AUCs for paired data can be statistically compared by estimating the covariance matrix for the AUCs, based on the general theory of U-statistics, and then constructing a large-sample test in the usual way. The implementation of this method, not freely available until now, provides the advantages and robustness of using a nonparametric approach for estimating AUCs in the case of paired datasets, accounting for the inherent correlation of this type of data. One limitation of this method that may be considered, as stated by DeLong *et al*. [14], is that the trapezoidal rule underestimates the true AUC when the variables take a small number of discrete values. Among the main features of our software are: 1) it is based on a non-parametric approach for the analysis of AUCs [14], 2) it uses a simple input format, 3) it can plot multiple ROC curves simultaneously, 4) the output data is compact, simple and can be exported for further analysis with other statistical tools; and 5) it generates a human-readable report in PDF format, which is useful for a fast initial inspection of the results.

It is worth noting that a freely available computer program for Windows, though still in its beta version, is DBM MRMC 2.1 [22]. This software is an extended version of a previous package, LABMRMC [23-28], which allows users to compare AUCs using the jackknife method. DBM MRMC 2.1 provides, among other functionalities, statistical analysis of the AUC computed by the trapezoidal method, which is equivalent to the AUC computed with the Mann-Whitney U-statistic. The program uses ANOVA methods together with jackknifing [23,25,26] (instead of the Delong method used by our program) to assess the statistical significance of the observed difference between two classifiers. Even though this software provides a wide range of alternatives in ROC fitting, measurement of ROC indexes and assessment of statistical significance for those indexes, DBM MRMC is still in its beta version at this moment and has the same drawbacks in terms of usability found in ROCKIT (these drawbacks are also present in LABMRMC package), such as the input/output handling, lack of automated options for fast analysis of many classifiers and lack of straightforward plotting of results.

## Software Implementation

The optimal threshold (OT) for each classifier is defined after the ROC analysis is performed and consists in the score value that leads to the maximal accuracy of classification. The assessment of the statistical significance of the differences of the AUCs between two classifiers is implemented as previously described [14]. Briefly, suppose that *R *tests are applied on the same *N *individuals, which can be classified as positive or negative. Suppose that *m *of these individuals are actually positive and *n *are actually negative (*m*+*n *= *N*), and that positive individuals tend to have greater values than negative individuals. If we let $\left\{X_{i}^{r}\right\}$ and $\left\{Y_{j}^{r}\right\}$ be the sets of outcome values on the *r*-th test that correspond to the positive and negative individuals, respectively (*i *= 1,...,*m*; *j *= 1,...,*n*; 1 ≤ *r *≤ *R*), the AUC for each classifier is computed with the Mann-Whitney U-statistic for comparing distributions of values from two samples, as follows:

$$\begin{array}{ccc} {\hat{\theta }}^{r}=\frac{1}{mn}\sum_{j=1}^{n}\sum_{i=1}^{m}\Psi \left(X_{i}^{r},Y_{j}^{r}\right) & \text{with} & \Psi \left(X,Y\right)=\{\begin{array}{c} 1;Y<X\\ \frac{1}{2};Y=X\\ 0;Y>X \end{array} \end{array}$$

The theory on generalized U-statistics allows us to obtain an estimated covariance matrix for two or more AUC estimates of correlated ROC curves; this *R *× *R *matrix is computed as follows:

$$S=\frac{1}{m}S_{10}+\frac{1}{n}S_{01}$$

where the (*r*_1_, *r*_2_)th element of *S*_10 _is given by

$${\left[S10\right]}_{r_{1},r_{2}}=\frac{1}{m-1}\cdot \sum_{i=1}^{m}\left[\frac{1}{n}\sum_{j=1}^{n}\Psi \left(X_{i}^{r_{1}},Y_{j}^{r_{1}}\right)-{\hat{\theta }}^{r_{1}}\right]\cdot \left[\frac{1}{n}\sum_{j=1}^{n}\Psi \left(X_{i}^{r_{2}},Y_{j}^{r_{2}}\right)-{\hat{\theta }}^{r_{2}}\right]$$

and similarly

$${\left[S01\right]}_{r_{1},r_{2}}=\frac{1}{n-1}\cdot \sum_{j=1}^{n}\left[\frac{1}{m}\sum_{i=1}^{m}\Psi \left(X_{i}^{r_{1}},Y_{j}^{r_{1}}\right)-{\hat{\theta }}^{r_{1}}\right]\cdot \left[\frac{1}{m}\sum_{i=1}^{m}\Psi \left(X_{i}^{r_{2}},Y_{j}^{r_{2}}\right)-{\hat{\theta }}^{r_{2}}\right]$$

For further explanation on how to get these estimates, please refer to reference [14]. Accounting for correlation between ROC curves is a necessary step for paired data where two classifiers are used on the same subjects. For balanced unpaired data the off-diagonal elements of S are set to zero since the AUCs are not correlated. Consequently, the covariance matrix S is used to compute the following chi-squared statistic for testing if there is a difference between two or more classifiers:

$${\left(L\hat{\theta }\right)}^{\prime }{\left[LSL^{\prime }\right]}^{-1}L\hat{\theta }$$

Here, $\hat{\theta }$ is the vector of AUC estimates and L is a suitable contrast matrix (*ie*. H0: *Lθ *= 0, where 0 is the zero matrix). The statistic follows a chi-squared distribution with rank(L) degrees of freedom under the null hypothesis of no difference between classifier AUCs. For a pair of classifiers the statistic reduces to

$$\frac{{\left(L\hat{\theta }\right)}^{2}}{\left(LSL^{\prime }\right)}$$

and a (1 - *α*)100% confidence interval is given by

$$L\hat{\theta }\pm z_{\alpha /2}\sqrt{LSL^{\prime }}$$

This particular software implementation and its successful application have been recently validated by us through the comparison to other software [29-31]. In these studies hundreds of classifiers for the prediction of errors in protein structures were assessed, and the results of statistical significance obtained with StAR software were consistent with those from ROCKIT.

## Software Description

The input of the software simply consists of two data files containing the positive and negative subjects, as defined by the user. It is important to be aware of the definition used for the negative and positive data, since the meaning or interpretation of false positives and true positives reported will depend on this definition. Each input file must have a multi-column format, where a given column contains the obtained scores from a specific classifier for all subjects tested. Additional input parameters that do not affect the results of the calculations are optional and include a job name and the possibility of getting the display of the classifiers sorted by decreasing AUC values, among others. Detailed on-line help about the required format for the input files is provided.

An initial summarized report with the results of the calculations for each classifier is given in a table that contains a variable number of columns, which correspond to the following in the most extended output case: 1) Sequential number of each classifier; 2) selection option of each classifier to perform further analysis (by default, all classifiers are selected); 3) description name or identification code of each classifier; 4) AUC of each classifier; 5) a plus sign ('+') indicating if the classifier score has been inverted in order to force the AUC greater or equal than 0.5, 6) maximal accuracy of each classifier (*ie*. obtained at an optimal classification threshold that is estimated after the ROC analysis); 7) optimal classification threshold (*ie*. the score value that, when used as a classification threshold, leads to the maximal accuracy); 8) false positive rate obtained at the optimal classification threshold; 9) true positive rate obtained at the optimal classification threshold; 10) total number of negative subjects evaluated; and 11) total number of positive subjects evaluated. Online description is provided for each field in this table.

Additionally, six additional actions on the provided data are available for further analysis. First, the user can plot the ROC curves for the selected classifiers. Some graphic display options or changes to the plots are available. Second, the estimated covariance matrix and the p-value of the global test for a difference between any of the classifiers is displayed. Third, the difference of any two classifiers provided can be assessed at a given significance level, which by default is set to 0.05, but it can be modified by the user. Fourth, for each pairwise comparison of the classifiers, the software reports the confidence intervals at a given confidence coefficient. Fifth, a human-readable report in PDF format that summarizes the results of the analysis can be generated. Finally, several files containing the detailed results from the analysis performed by the user at the selected significance level can be downloaded. These include the ROC plot points for each classifier, the estimated covariance matrix, a table containing the p-value and confidence interval of the AUC difference observed for each pairwise comparison of classifiers with color coding of the p-value used to indicate if the difference was significant.

The standalone version of this software is also released for the Linux operating system. The Linux version offers the same capabilities of the web server, but without the graphic display and the interactive options. A detailed tutorial that describes how to use the software is available at the server web site.

## Availability and Requirements

**Project name: **StAR: Statistical Comparison of ROC Curves.

**Project homepage: **

**Operating system(s): **any (web server version), Linux (standalone version).

**Programming language: **C++, PHP, PERL.

**Other requirements: **none

**License: **none

**Any restrictions to use by non-academics: **none

## List of abbreviations used

ROC: Receiver operating characteristic; AUC: Area under the ROC curve; OT: Optimal threshold; PDF: Portable document format; StAR: Statistical analysis of ROC curves.

## Authors' contributions

IAV and TN developed and implemented the core computer programs. EF and AWS wrote some additional scripts and tested the software. FM supervised this project and wrote the manuscript with the help of IAV. All authors read and approved the final version.
